# Interaction Effects of the Leu162Val PPAR****α**** and Pro12Ala PPAR****γ****2 Gene Variants with Renal Function in Metabolic Syndrome Population

**DOI:** 10.1155/2013/329862

**Published:** 2013-04-04

**Authors:** Sarraj Mohamed Youssef, Najah Mohamed, Slimani Afef, Ben Hamda Khaldoun, Neffati Fadoua, Najjar Mohamed Fadhel, Slimane Mohamed Naceur

**Affiliations:** ^1^Research Unit 05/UR/09-12: Genetic and Biological Factors of Atherosclerosis, Medicine Faculty, University of Monastir, Street Fatouma Bourguiba, 5000 Monastir, Tunisia; ^2^Department of Cardiology of the Fattouma Bourguiba University Hospital of Monastir, Tunisia; ^3^Laboratory of Biochemistry and Toxicology of the Fattouma Bourguiba University Hospital of Monastir, Tunisia

## Abstract

Leu162Val PPAR**α** and Pro12Ala PPAR**γ**2 were investigated for their individual and their interactive impact on MS and renal functionality (RF). 522 subjects were investigated for biochemical and anthropometric measurements. The diagnosis of MS was based on the IDF definition (2009). The HOMA 2 was used to determine HOMA-**β**, HOMA-S and HOMA-IR from FPG and FPI concentrations. RF was assessed by estimating the GFR. PCR-RFLP was performed for DNA genotyping. Allele frequencies were 0.845 for Pro and 0.155 for Ala, and were 0.915 for Leu and 0.085 for Val. We showed that carriers of the PPAR**α** Val 162 allele had lower urea, UA and higher GFR compared to those homozygous for the Leu162 allele. Subjects carried by PPAR**γ**2Ala allele had similar results. They also had reduced FPG, FPI and HOMA-IR, and elevated HOMA-**β** and HOMA-S compared to those homozygous for the Pro allele. Subjects were divided into 4 groups according to the combinations of genetic alleles of the 2 polymorphisms. Subjects carrying the Leu/Val with an Ala allele had lower FPG, PPI, HOMA-IR, urea, UA levels, higher HOMA-**β**, HOMA-S and GFR than different genotype combinations. Leu162Val PPAR**α** and Pro12Ala PPAR**γ**2 can interact with each other to modulate glucose and insulin homeostasis and expand their association with overall better RF.

## 1. Introduction

Metabolic syndrome (MS) is a complex disorder characterized by the clustering of several metabolic diseases such as abdominal obesity, insulin resistance (IR), elevated plasma triglycerides level (TG), low high density lipoprotein cholesterol (cHDL), high blood pressure, and altered glucose homeostasis [1]. Environmental factors such as low physical activity and inappropriate dietary habits are strong determinants of the MS. In addition, genetic factors also contribute to the individual susceptibility to MS [2].

All components of the MS have individually been associated with the incidence and progression of chronic kidney diseases (CKDs). The mechanisms and impacts of hypertensive and diabetic injuries, the two major etiologies of CKD in the world, have been well studied and described [3–5]. Several observational studies found that individuals with the MS are at increased risk for presenting renal manifestations, namely, microalbuminuria and decreased glomerular filtration rate (GFR). In fact, epidemiological studies have linked MS with an increased risk for microalbuminuria, an early marker of glomerular injury and endothelial dysfunction [6–8].

Peroxisome proliferator-activated receptors (PPARs) are nuclear hormone receptors. They are ligand-dependent intracellular proteins that stimulate transcription of specific genes by binding to specific DNA sequences [9]. There are three PPAR subtypes, products of the distinct genes commonly designated as PPAR*α*, PPAR*γ*, and PPAR *β*/*δ* and expressed in various tissues [10–13]. In humans, renal PPAR*α* and PPAR*γ* isotypes are abundantly expressed [14, 15].

In this regard, two common polymorphisms affecting the amino acid sequence of the PPAR*α* and PPAR*γ*2 gene are relevant candidates, the Leu162Val PPAR*α* and Pro12Ala PPAR*γ*2. We, therefore, assessed the potential relationships of these polymorphisms variants for their individual effect as well as their interactive impact on MS and renal injuries.

## 2. Materials and Methods

### 2.1. Study Population

522 subjects undergoing routine control were investigated for biochemical, anthropometric, and clinical examination at the occupational medicine of the University Hospital of Monastir, Tunisia. All the subjects enrolled in this study were coming from central Tunisia and there were no consanguinity relationships among them. Participants gave their written informed consent prior to their participation. The study was approved by the ethical committee of the hospital.

### 2.2. Diagnostic Criteria for Metabolic Syndrome

The diagnosis of MS was based on the IDF and AHA/NHLBI definition, which requires the presence of at least three of the following criteria: the central (abdominal) obesity (defined as waist circumference (WC) ≥94 cm in men and ≥80 cm in women), the raised TG ≥1.70 mmol/L (drug treatment for elevated triglycerides is an alternate indicator), the reduced cHDL <1.04 mmol/L in men and <1.29 mmol/L in women (or specific treatment for this lipid abnormality), the elevated systolic blood pressure (SBP) ≥130 mmHg and/or diastolic blood pressure (DBP) ≥85 mmHg (antihypertensive drug treatment in a patient with a history of hypertension was an alternate indicator), and the elevated FPG ≥5.56 mmol/L or previously diagnosed type 2 diabetes [16].

### 2.3. Anthropometric Measurements

Height and weight were measured according to a standardized protocol in the study population, with subjects wearing light clothing and no shoes. Body mass index (BMI) was calculated by dividing weight in kilograms by height in square meters (kg/m^2^). The waist circumference was measured in the horizontal plane at the midpoint between the lowest rib and the iliac crest. Systolic blood pressure (SBP) and diastolic blood pressure (DBP) were measured to the nearest 5 mmHg with a mercury sphygmomanometer, with subjects in a supine position and having relaxed for 5 minutes.

### 2.4. Biochemical Analysis

The blood samples of the study population were collected in the morning after a 12-hour fasting period, heparinazed serum was immediately obtained by blood centrifugation at 4°C at 3000 rpm for 15 min. All analyses were carried out in biochemistry and toxicology laboratory of the Hospital using a Cobas 6000TM analyzer (Roche Diagnostics Mannheim, Germany). Serum total cholesterol (TC), triglycerides (TG), high density lipoprotein cholesterol (cHDL), low density lipoprotein cholesterol (cLDL), and Uric acid (UA) serum levels and fasting plasma glucose (FPG) were determined by using enzymatic techniques. Fasting plasma insulin (FPI) was measured by electrochemiluminescence immuno assay (ECLIA), and serum creatinine concentration was determined by the kinetic Jaffe method.

The computer model HOMA 2 was used to determine *β*-cell function (HOMA-*β*%), insulin sensitivity (HOMA-S%), and insulin resistance (HOMA-IR) from paired fasting glucose (mmol/L) and insulin (mIU/L) concentrations [17].

Renal function was assessed by estimating the GFR, with the Cockcroft and Gault formula:

(1) $$\begin{array}{c} \text{GFR}\;\left(\text{mL}/\min\right)=\frac{\left[140-\text{age}\;\left(\text{years}\right)\right]\;\times \text{BM}\;\left(\text{kg}\right)}{0.814\times \text{creatinine}\;\left(\mu \text{mol}/\text{L}\right)}. \end{array}$$

In female subjects, the result was multiplied by 0.85 [18].

### 2.5. Genetic Analysis

Genotyping was carried out on genomic DNA extracted from subjects' blood samples by salt fractionation. The primers used for polymerase chain reaction-restriction fragment length polymorphism (PCR-RFLP) from the PPAR *α* Leu 162 Val SNP were 5′-GACTCAAGCTGGTGTATGACAAGT-3′ as the forward primer and the reverse-mismatch primer 5′CGTTGTGTGACATCCCGACAGAAT-3′ with the mismatch nucleotide in the reverse primer underlined. A mixture of Taq polymerase 10X buffer 2.5 *μ*L, 0.2 *μ*L Taq DNA polymerase, 2 *μ*L dNTP, 10 pmol of each primer, and 6 *μ*L of template DNA was used at a total volume of 25 *μ*L, and the mixture was amplified in PCR equipment (TECHNE TC-312). The reaction was carried out using 30 cycles of predenaturation at 94°C for 3 min, denaturation at 94°C for 30 s, annealing at 58°C for 30 s, and elongation at 72°C for 5 min. Electrophoresis was conducted in 1% agarose gel to confirm the 117 bp PCR product, and the restriction was performed by using 8 U of a Hinf I enzyme at 37°C overnight. The restriction site was confirmed at 3% agarose gel. The Leu/Leu homozygote produced 1 fragment at 117 bp, the Leu/Val heterozygote produced 3 fragments at 117, 93, and 24 bp, and the Val/Val homozygote produced 2 fragments at 93 and 24 bp. The primers used for PCR-RFLP from the PPAR*γ*2 Pro 12 Ala SNP were 5′-CAAGCCCAGGTCCTTTCTGTG-3′ as the forward primer and 5′-AGTGAAGGAATCGCTTTCCG-3′ as the reverse primer. A mixture of Taq polymerase 10X buffer 2.5 *μ*L, 0.2 *μ*L Taq DNA polymerase, 2 *μ*L dNTP, 10 pmol of each primer, and 6 *μ*L of template DNA was used at a total volume of 25 *μ*L, and the mixture was amplified in PCR equipment (TECHNE TC-312). The reaction was carried out using 30 cycles of predenaturation at 94°C for 3 min, denaturation at 94°C for 30 s, annealing at 60°C for 30 s, and elongation at 72°C for 5 min. Electrophoresis was conducted in 1% agarose gel to confirm the 237 bp PCR product, and the restriction was performed by using 8 U of a HpaII enzyme at 37°C overnight. The restriction site was confirmed at 3% agarose gel. The Pro/Pro homozygote produced 2 fragments at 217 and 20 bp, the Pro/Ala heterozygote produced 3 fragments at 237, 217, and 20 bp, and the Ala/Ala homozygote produced 1 fragment at 237 bp, which did not splice when the HpaII restriction enzyme was used.

### 2.6. Statistical Analysis

Data were analyzed by SPSS 17.0 for Windows. Continuous results that satisfied a normal distribution are expressed as mean ± standard deviation (SD). Those results that provided abnormal distribution data are expressed as median and quartile and frequencies for qualitative variables. Comparisons among groups were assessed using the independent-sample *t*-test for quantitative variables and Pearson's chi-square test for qualitative variables. The one-way analysis of variance (ANOVA) method was used to compare differences between genotype groups. Pearson's chi-square test (*χ* ^2^) was used to compare the genotype prevalence between different groups. The Hardy-Weinberg equilibrium was performed using the *χ* ^2^ test. A two-sided *P* < 0.05 was considered as statistically significant.

## 3. Results

Among the 522 subjects who were enrolled, 258 presented metabolic syndrome (SM^+^) and 264 were without (MS^−^).  Table 1 shows that SM^+^ subjects have higher BMI, WC, SBP, DBP, TG, cLDL, TC, FPG, and FPI and reduced cHDL compared to subjects MS^−^. We also noted elevated IR and reduced HOMA *β* and HOMA S and higher creatinine, urea, and UA plasma level and reduced GFR in SM^+^ group.

The Pro/Pro genotype was present in 70.69% (369 subjects); Pro/Ala genotype in 27.59% (144 subjects) and Ala/Ala genotype was present in 1.72% (9 subjects) of 522 subjects. Allele frequencies were 0.845 for Pro allele and 0.155 for Ala allele. Allele frequency of the two genotypes satisfied the Hardy-Weinberg equilibrium. The Leu/Leu genotype was present in 84.48% (441 subjects), Leu/Val genotype in 14.17% (74 subjects), and Val/Val genotype in 1.35% (7 subjects) of 522 subjects. Allele frequencies were 0.916 for Leu allele and 0.084 for Val allele. Allele frequency of the two genotypes satisfied the Hardy-Weinberg equilibrium. The allelic frequency of Ala allele was significantly (*P* = 0.005) lower in MS^+^ group (0.124%) than in MS^−^ group (0.186). Also, the frequency of Val allele was significantly (*P* = 0.006) lower in MS^+^ group (0.061) than in MS^−^ group (0.108). Subjects with the Ala allele had a decreased risk for MS (odds ratio (OR) = 0.621, 95% CI (0.442–0.874)). The Val allele decreases the risk of MS (OR = 0.528, 95% CI (0.335–0.833)) (Table 2).

The independent effects of each polymorphism on anthropometric and biochemical characteristics-related variables are presented in Table 3. There were no differences among the genotypes in terms of age, BMI, blood pressure, TG, TC, cHDL, and cLDL either in X/Ala (Pro/Ala and Ala/Ala) PPAR*γ*2 or X/Val (Leu/Val and Val/Val) PPAR*α*. Subjects with Pro/Pro had a significantly higher FPG, FPI, and HOMA-IR. In parallel, creatinine, urea, and uric acid serum levels were found to be elevated in these Pro/Pro subjects. In addition, Pro/Pro subjects also display reduced HOMA-*β* and HOMA-S together with decreased GFR compared to X/Ala (Pro/Ala and Ala/Ala) subjects. Furthermore, subjects with Leu/Leu have only a significantly reduced HOMA-*β* and elevated creatinine, urea, and uric acid serum levels and a decreased GFR compared to X/Val (Leu/Val and Val/Val) subjects.

To check if the effects of the SNPs are associated with MS, subjects were divided into people displaying metabolic syndrome (SM^+^) or not (MS^−^) (Tables 4 and 5). For Pro12Ala PPAR*γ*2 gene in the SM^+^ group, Ala allele carriers had higher HOMA-*β*, HOMA-S and GFR, and decreased FPG, FPI, HOMA-IR, creatinine, and urea serum levels compared to non-Ala allele carriers. For Leu162Val PPAR*α* gene, both groups did not show any significant difference on the measures.

To check if the effects of the SNPs are associated with age, we divided the subjects into two groups. The first group had subjects who were younger than 60 years old, and the second group had subjects that were 60 years old and more; here we compared the parameters related to MS between these two groups (Tables 6 and 7). For Pro12Ala PPAR*γ*2 gene, in the group aged <60 years old, Ala allele carriers had higher HOMA-*β* and HOMA-S and decreased FPG, FPI, HOMA-IR, creatinine, and urea serum levels than non-Ala allele carriers. For Pro12Ala PPAR*γ*2 gene, in the group aged ≥60 years old, Ala allele carriers had higher HOMA-S and GFR and decreased FPI and HOMA-IR than non-Ala allele carriers. For Leu162Val PPAR*α* gene, both groups showed significantly higher HOMA-*β* and reduced FPG levels in Val-carriers than Pro-carriers.

To check if the effects of the SNPs are associated with BMI, we divided the subjects into three groups, a lean group (BMI < 25 kg/m^2^), an overweight group (BMI ≥ 25 kg/m^2^ and BMI ≤ 30 kg/m^2^), and obese group (BMI > 30 kg/m^2^). We compared the genotype and allele frequency among the three groups by using Pearson's chi-square test (*χ* ^2^). There was no difference among the three groups in terms of both polymorphisms (*P* = 0.063, 0.902). Further analysis by gender was conducted and same result was achieved by both males and females. There was no difference found in terms of Leu162Val and Pro 12 Ala PPAR*γ*2 in genotype of both polymorphisms (*P* = 0.224, 0.889).

In order to evaluate the interaction between Leu162Val PPAR*α* and Pro12Ala PPAR*γ*2 polymorphisms, we divided the subjects into four groups according to the combination of the 2 genotypes from each SNP, that is, Leu/Leu PPAR*α* with Pro/Pro PPAR*γ*2 genotype (*n* = 329), Leu/Leu PPAR*α* with an Ala allele (*n* = 112), Leu/Val PPAR*α* with Pro/Pro PPAR*γ*2 (*n* = 40), and Leu/Val PPAR*α* with an Ala allele (*n* = 41), and compared the study parameters among the groups. There were no differences in the anthropometric measurements and lipid profile variables among the different combination groups of the polymorphisms. The Leu/Val genotype carries with an Ala allele group had significantly reduced FPG, FBI, HOMA-IR, urea, and UA levels and elevated HOMA-S, HOMA-*β*, and GFR compared to the different combination groups (Table 8).

## 4. Discussion

MS is a complex disorder resulting from the interaction between genetic and environmental factors. A major part of our study has focused on the genetics of PPAR*α* and PPAR*γ*2 polymorphisms. Understanding the genetics of these polymorphisms is not only important because it is associated with the MS, but also it has been recently recognized to be related to renal function [19–22]. The present study investigated the independent effect of Leu162Val PPAR*α* and Pro12Ala PPAR*γ*2 polymorphisms as well as their impact on glucose, insulin, HOMA index, urea, UA, and GFR.

We showed that carriers of the PPAR*α* Val162 allele had lower urea, UA, and raised GFR compared to those homozygous for the Leu162 allele. Also, subjects carrying the PPAR*γ*2 Ala allele had the same results. In addition, they had reduced FPG, FPI, and HOMA-IR and elevated HOMA-*β* and HOMA-S. The Leu/Val genotype carriers with an Ala allele group had lower FPG, PPI, HOMA-IR, urea, and UA levels and higher HOMA-*β*, HOMA-S, and GFR than other different genotype combinations. Thus, the effect of one allele in one gene seems to depend upon the presence of another allele in a second gene.

The Leu162Val PPAR*α* and Pro12Ala PPAR*γ*2 polymorphisms have opposite effects on the transcriptional activity of their respective receptors. Indeed, the Ala12 allele results in a less active form of PPAR*γ*2, while the Val162 allele results in a more active form of PPAR*α* [23–25]. However, the observation that the PPAR*γ*2 Ala12 allele mediates its lowering effect only on a PPAR*α* genetic background complicates the explanation. It demonstrated that the PPAR*γ*2 Ala12 allele was associated with greater insulin sensitivity [25]. Similarly, the Ala12 allele in PPAR*γ*2 attenuates the effect of the PPAR*α* Val162 allele on glucose and insulin homeostasis [26]. Genetic variation in PPAR*γ* coactivator-1, which also coactivates PPAR*α*, influences the insulin secretory response [27].

Many studies found a significant association between MS and CKD and consistently demonstrated an increased risk parallel to the number of MS traits [28–30]. The association between MS and renal damage is, in part, explained by hypertension and impaired glucose metabolism. However, the underlying mechanisms include an increasing body mass, insulin resistance, inflammation, renal endothelial dysfunction, oxidative stress, and altered renal haemodynamics, activation of the renin-angiotensin-aldosterone system and sympathetic nervous system, and dietary factors [31].

Urine analysis and blood biochemistry have been of great help in the assessment of renal function. Uric acid is an end product of purine (a component of nucleic acids and nucleoproteins) metabolism; urea is an end product of protein metabolism and the creatinine is derived from the creatine and is a waste product. The major cause of increased levels of plasma creatinine, urea, and uric acid is the poor clearance of these substances by the kidneys rather than excessive production. Insulin may induce renal fibrosis by stimulating mesangial cells and proximal tubule cells to produce tumor growth factor *β* (TGF-*β*) [32, 33].

Insulin stimulates the production of insulin-like growth factory 1 (IGF-1) by vascular smooth muscle cells and other cell types, which have been implicated in the development of diabetic kidney disease [34]. IGF-1 increases the activity of connective tissue growth factor, a cytokine that has profibrogenic actions on renal tubular cells and interstitial fibroblasts. In addition, IGF-1 decreases the activity of matrix metalloproteinase-2, an enzyme responsible for extracellular matrix degradation, thereby promoting extracellular matrix expansion and renal fibrosis [35, 36]. Additionally, IR promotes sodium and UA reabsorption resulting in salt-sensitive hypertension and hyperuricemia [37].

Insulin resistance and the release of inflammatory cytokines induce mesangial expansion, basement membrane thickening, podocytopathy, and loss of slit pore diaphragm integrity leading to the so-called obesity-related glomerulopathy [38, 39]. In accordance with the results of other studies, expression of PPAR*α* in glomerular mesangial cells has also been reported. Thus, it is likely that PPAR*α* activation in mesangial cells could block TGF-*β* signalling pathway by attenuating glomerular matrix proliferation. Therefore, it is likely that PPAR*α* activation may facilitate albumin reabsorption and degradation in the nephron segment [40, 41]. Bossé et al. observed a deleterious effect of the PPAR*α* Val162 allele on glucose and insulin levels during a glucose challenge but suggested that Leu162Val PPAR*α* and Pro12Ala PPAR*γ*2 polymorphisms interact with each other to modulate some features of glucose and insulin homeostasis [42]. Moreover, it was found that the Ala-allele is associated with enhanced decline in GFR and predicts end-stage renal disease (ESRD) and all-cause mortality in patients with nephropathy [43].

Taken together, these observations may partly explain the synergetic effect of Leu162Val PPAR*α* and Pro12Ala PPAR*γ*2 polymorphisms on MS and renal injuries.

## 5. Conclusion

We suggest that Leu162Val PPAR*α* and Pro12Ala PPAR*γ*2 polymorphisms can interact with each other to modulate glucose and insulin homeostasis and expand their association with the overall renal function. However, a replication of this study is required before a firm conclusion can be reached.

## Figures and Tables

**Table 1:** Anthropometric and biochemical characteristics of study population.

Variables	MS^−^ (*n* = 264)	MS^+^ (*n* = 258)	*P *
Age (years)	41.0 (29.0–51.7)	38.0 (29.0–54.0)	0.695
Gender M/F (%)	137/127 (51.9/48.1)	128/130 (49.6/50.4)	0.602
Diabetes (*n* (%))	31 (11.7)	71 (27.9)	<0.001
Hypertension (*n* (%))	36 (13.6)	101 (39.7)	<0.001
SBP (mmHg)	120 (115–125)	140 (130–150)	<0.001
DBP (mmHg)	80 (75–80)	85 (80–90)	<0.001
BMI (kg/m^2^)	24.5 (23.7–25.9)	27.7 (26.4–29.4)	<0.001
Men WC (cm)	93 (89–95)	98 (96–101)	<0.001
Women WC (cm)	79 (77–86)	93 (90–98)	<0.001
TG (mmol/L)	1.07 ± 0.44	1.91 ± 0.71	<0.001
TC (mmol/L)	4.51 (4.12–4.99)	5.15 (4.24–5.78)	<0.001
cLDL (mmol/L)	2.92 (2.52–3.31)	3.36 (2.76–3.95)	<0.001
Men cHDL (mmol/L)	1.10 (1.02–1.16)	0.84 (0.74–0.92)	<0.001
Women cHDL (mmol/L)	1.45 (1.34–1.53)	1.10 (0.89–1.26)	<0.001
FPG (mmol/L)	4.94 ± 0.95	6.96 ± 1.78	<0.001
FPI (mIU/L)	7.12 (5.97–8.61)	12.73 (9.59–13.22)	<0.001
HOMA-*β*%	97 (88–109)	72 (52–94)	<0.001
HOMA-S%	110 (88–130)	56 (53–78)	<0.001
HOMA-IR	0.9 (0.8–1.1)	1.8 (1.3–1.9)	<0.001
Cr (*μ*mol/L)	87.5 ± 31	111.4 ± 46.4	<0.001
Urea (mmol/L)	4.46 ± 2.63	7.72 ± 4.82	<0.001
UA (*μ*mol/L)	267.5 (213.5–307.7)	358 (285–4.28)	<0.001
GFR (mL/min)	92.9 (78.8–107.4)	85.6 (61.9–106.3)	0.004

MS^−^: without metabolic syndrome; MS^+^: with metabolic syndrome, SBP: systolic blood pressure, DBP: diastolic blood pressure; BMI: body mass index; WC: waist circumference; TG: triglycerides; TC: total cholesterol; cHDL: high density lipoprotein cholesterol; cLDL: low density lipoprotein cholesterol; FPG: fasting plasma glucose; FPI: fasting plasma insulin; HOMA-*β*%: %  *β*-cell function; HOMA-S%: % cell insulin sensitivity; HOMA-IR: insulin resistance; Cr: creatinine; UA: uric acid; GFR: glomerular filtration rate.

**Table 2:** Leu162Val PPAR*α* and Pro12Ala PPAR*γ*2 genotype and allele distribution.

	Total (522)	MS^−^ (264)	MS^+^ (258)	*P *	OR (95% CI)
Pro12 Ala PPAR*γ*2					

Allele					
Pro (*n* (%))	882 (84.5)^a^	430 (81.4)	452 (87.6)	0.006	1.610 (1.144–2.265)
Ala (*n* (%))	162 (15.5)	98 (18.6)	64 (12.4)	0.006	0.621 (0.442–0.874)
Genotype					
Pro/Pro (*n* (%))	369 (70.69)	174 (65.91)	195 (75.58)	0.015	1.601 (1.093–2.344)
Pro/Ala (*n* (%))	144 (27.59)	82 (31.06)	62 (24.03)	0.046	0.675 (0.458–0.995)
Ala/Ala (*n* (%))	9 (1.72)	8 (3.03)	1(0.39)	0.013	0.112 (0.014–0.901)

Leu162Val PPAR*α*					

Allele					
Leu (*n* (%))	956 (91.6)	471 (89.2)	485 (93.9)	0.005	1.893 (1.201–2.985)
Val (*n* (%))	88 (8.4)	57 (10.8)	31 (6.1)	0.005	0.528 (0.335–0.833)
Genotype					
Leu/Leu (*n* (%))	441 (84.48)	213 (80.68)	228 (88.37)	0.015	1.820 (1.117–2.985)
Leu/Val (*n* (%))	74 (14.17)	45 (17.05)	29 (11.24)	0.046	0.602 (0.364–0.995)
Val/Val (*n* (%))	7 (1.35)	6 (2.27)	1 (0.39)	0.049	0.156 (0.019–1.304)

^a^Number (% of total); SM^−^: without metabolic syndrome; SM^+^: with metabolic syndrome; OR: odds ratio; CI: confidence interval.

**Table 3:** Anthropometric, HOMA index, and biochemical characteristics of subjects with different groups of genotype of the Leu162Val PPAR*α* and Pro12Ala PPAR*γ*2 polymorphisms.

Parameters	Pro12Ala PPAR*γ*2			Leu162Val PPAR*α*		
	P/P (*n* = 369)	X/A (*n* =153)	*P *	L/L (*n* = 441)	X/V (*n* = 81)	*P *
Age (years)	41 (29–53)	38 (28–49)	0.088	39 (29–53)	38 (29–48)	0.177
SBP (mmHg)	125 (120–140)	125 (120–135)	0.102	125 (120–140)	130 (120–135)	0.854
DBP (mmHg)	80 (80–85)	80 (80–85)	0.127	80 (80–85)	80 (80–85)	0.465
BMI (kg/m^2^)	26.4 (24.4–28.3)	25.6 (24.1–27.9)	0.108	25.9 (24.3–28.2)	26.6 (24.4–29.1)	0.201
WC (cm)	93.0 (85.0–98.0)	93.0 (81.5–97)	0.404	93.0 (83.0–97.0)	94.0 (86.5–98.5)	0.076
TG (mmol/L)	1.51 ± 0.71	1.43 ± 0.76	0.186	1.50 ± 0.72	1.44 ± 0.72	0.497
TC (mmol/L)	4.73 (4.19–5.49)	4.71 (4.11–5.27)	0.271	4.72 (4.19–5.45)	4.71 (4.12–5.41)	0.526
cLDL (mmol/L)	3.09 (2.61–3.71)	3.00 (2.62–3.67)	0.513	3.07 (2.62–3.73)	3.09 (2.57–3.63)	0.410
cHDL (mmol/L)	1.12 (0.88–1.27)	1.09 (0.90–1.34)	0.368	1.11 (0.88–1.29)	1.12 (0.92–1.33)	0.475
FPG (mmol/L)	6.14 ± 1.77	5.45 ± 1.57	<0.001	6.05 ± 1.79	5.36 ± 1.30	0.001
FPI (mIU/L)	9.83 (7.12–12.88)	7.85 (6.16–11.22)	<0.001	9.23 (6.92–12.83)	8.91 (6.65–11.95)	0.139
HOMA-*β*%	86 (64–100)	95 (82–105)	<0.001	88 (67–99)	95 (80–119)	<0.001
HOMA-S%	77 (54–109)	102 (68–126)	<0.001	80 (55–112)	85 (62–118)	0.043
HOMA-IR	1.3 (0.9–1.8)	1.0 (0.8–1.5)	<0.001	1.2 (0.9–1.8)	1.2 (0.9–1.6)	0.067
Cr (*μ*mol/L)	102.1 ± 45.8	92.5 ± 25.2	0.016	99.6 ± 41.6	97.9 ± 38.2	0.749
Urea (mmol/L)	6.45 ± 4.33	5.17 ± 2.34	0.001	6.29 ± 4.13	4.89 ± 1.83	0.003
UA (*μ*mol/L)	307 (248–385)	292 (234–350)	0.036	306 (247–382)	292 (233–341)	0.042
GFR (mL/min)	89.5 (72.7–105.1)	93.8 (71.6–111.3)	0.004	89.0 (71.5–104.9)	102.6 (76.1–113.5)	0.002

P: Pro; A: Ala; L: Leu; V: Val; X/A: Pro/Ala and Ala/Ala; X/V: Leu/Val and Val/Val.

SBP: systolic blood pressure, DBP: diastolic blood pressure; BMI: body mass index; WC: waist circumference; TG: triglycerides; TC: total cholesterol; cHDL: high density lipoprotein cholesterol; cLDL: low density lipoprotein cholesterol; FPG: fasting plasma glucose; FPI: fasting plasma insulin; HOMA-*β*%: %  *β*-cell function; HOMA-S%: % cell insulin sensitivity; HOMA-IR: insulin resistance; Cr: creatinine; UA: uric acid; GFR: glomerular filtration rate.

**Table 4:** Comparison of anthropometric, HOMA index, and biochemical characteristics of subjects between different groups of Pro12Ala PPAR*γ*2 genotypes in two subdivided groups according to metabolic syndrome.

Parameters	SM^−^			SM^+^		
	Pro12Ala PPAR*γ*2			Pro12Ala PPAR*γ*2		
	P/P (*n* = 174)	X/A (*n* = 90)	*P *	P/P (*n* = 195)	X/A (*n* = 63)	*P *
Age (years)	42 (29–53)	38 (28–49)	0.455	39 (29–56)	37 (28–49)	0.103
SBP (mmHg)	120 (115–125)	120 (115–125)	0.708	140 (130–150)	140 (130–145)	0.675
DBP (mmHg)	80 (75–80)	80 (70–80)	0.277	85 (80–90)	85 (80–90)	0.965
BMI (kg/m^2^)	24.5 (23.8–25.9)	24.4 (23.6–25.7)	0.228	27.7 (26.3–29.4)	27.6 (26.5–29.3)	0.940
WC (cm)	87 (79–93)	90 (79–93)	0.758	96 (92–99)	96 (93–99)	0.997
TG (mmol/L)	1.07 ± 0.43	1.07 ± 0.43	0.942	1.91 ± 0.67	1.92 ± 0.84	0.964
TC (mmol/L)	4.51 (4.15–4.85)	4.62 (4.02–5.17)	0.633	5.18 (4.27–5.78)	4.81 (4.19–5.59)	0.190
cLDL (mmol/L)	2.90 (2.54–3.29)	2.98 (2.40–3.53)	0.729	3.48 (2.76–4.10)	3.24 (2.85–3.85)	0.499
cHDL (mmol/L)	1.18 (1.11–1.43)	1.19 (1.04–1.46)	0.868	0.91 (0.79–1.17)	0.92 (0.77–1.14)	0.925
FPG (mmol/L)	4.94 ± 1.09	4.95 ± 0.87	0.946	7.21 ± 1.67	6.16 ± 1.86	<0.001
FPI (mIU/L)	7.13 (6.14–8.65)	6.93 (5.61–8.58)	0.277	12.83 (10.76–13.29)	10.58 (7.92–12.98)	<0.001
HOMA-*β*%	96 (87–111)	97 (88–106)	0.698	68 (52–88)	86 (71–101)	<0.001
HOMA-S%	108 (87–128)	113 (89–140)	0.270	56 (53–66)	70 (55–103)	<0.001
HOMA-IR	0.9 (0.8–1.2)	0.9 (0.7–1.1)	0.282	1.8 (1.5–1.9)	1.4 (1.0–1.8)	<0.001
Cr (*μ*mol/L)	87.29 ± 36.16	87.87 ± 25.2	0.579	115.35 ± 49.51	99.30 ± 32.42	0.017
Urea (mmol/L)	4.52 ± 3.05	4.35 ± 17.24	0.619	6.33 ± 2.78	8.16 ± 4.58	0.003
UA (*μ*mol/L)	268 (210–313)	266 (215–306)	0.996	358 (285–437)	348 (278–396)	0.062
GFR (mL/min)	93.4 (82.3–106.4)	91.5 (70.5–109.1)	0.192	84.0 (58.8–104.5)	98.8 (78.8–113.8)	0.001

P: Pro; A: Ala; X/A: Pro/Ala and Ala/Ala.

SBP: systolic blood pressure, DBP: diastolic blood pressure; BMI: body mass index; WC: waist circumference; TG: triglycerides; TC: total cholesterol; cHDL: high density lipoprotein cholesterol; cLDL: low density lipoprotein cholesterol; FPG: fasting plasma glucose; FPI: fasting plasma insulin; HOMA-*β*%: %  *β*-cell function; HOMA-S%: % cell insulin sensitivity; HOMA-IR: insulin resistance; Cr: creatinine; UA: uric acid; GFR: glomerular filtration rate.

**Table 5:** Comparison of anthropometric, HOMA index, and biochemical characteristics of subjects between different groups of Leu162Val PPAR*α* genotypes in two subdivided groups according to metabolic syndrome.

Parameters	SM^−^			SM^+^		
	Leu162Val PPAR*α*			Leu162Val PPAR*α*		
	L/L (*n* = 213 )	X/V (*n* = 51)	*P *	L/L (*n* = 228 )	X/V (*n* = 30)	*P *
Age (years)	42 (29–52)	38 (32–49)	0.566	39 (29–56)	36 (29–45)	0.898
SBP (mmHg)	120 (115–120)	120 (120–130)	0.073	140 (130–150)	135 (130–150)	0.300
DBP (mmHg)	80 (75–80)	80 (75–80)	0.260	85 (80–90)	85 (80–90)	0.090
BMI (kg/m^2^)	24.5 (23.7–25.8)	25.0 (23.9–28.1)	0.172	27.7 (26.4–29.4)	27.6 (26.5–29.4)	0.400
WC (cm)	86 (79–93)	93 (84–97)	0.658	96 (92–99)	96 (93–100)	0.615
TG (mmol/L)	1.06 ± 0.44	1.09 ± 0.42	0.691	1.90 ± 0.70	2.02 ± 0.77	0.382
TC (mmol/L)	4.51 (4.14–4.93)	4.53 (3.90–5.06)	0.659	5.15 (4.22–5.78)	5.12 (4.23–5.78)	0.330
cLDL (mmol/L)	2.92 (2.52–3.31)	2.97 (2.31–3.40)	0.717	3.42 (2.76–4.03)	3.30 (2.83–3.85)	0.351
cHDL (mmol/L)	1.19 (1.10–1.46)	1.16 (1.04–1.39)	0.777	0.90 (0.79–1.14)	0.95 (0.82–1.28)	0.935
FPG (mmol/L)	4.94 ± 0.96	4.96 ± 0.88	0.846	7.08 ± 1.76	6.02 ± 1.60	0.002
FPI (mIU/L)	7.06 (5.86–8.25)	7.48 (6.17–10.04)	0.626	12.77 (10.09–13.25)	11.26 (7.85–13.03)	0.879
HOMA-*β*%	97 (88–105)	100 (86–121)	0.283	69 (52–93)	85 (66–106)	0.219
HOMA-S%	111 (95–134)	103 (75–126)	0.602	56 (53–73)	64 (56–99)	0.564
HOMA-IR	0.9 (0.8–1.1)	1.0 (0.8–1.3)	0.761	1.8 (1.4–1.9)	1.6 (1.0–1.8)	0.457
Cr (*μ*mol/L)	86.3 ± 32.6	92.3 ± 22.5	0.216	111.9 ± 45.2	107.6 ± 54.7	0.631
Urea (mmol/L)	4.55 ± 2.87	4.12 ± 1.17	0.303	7.92 ± 4.46	6.02 ± 2.01	0.039
UA (*μ*mol/L)	268 (206–313)	250 (223–302)	0.752	358 (285–433)	348 (288–398)	0.847
GFR (mL/min)	91.7 (79.4–105.8)	99.1 (71.6–109.4)	0.302	84.5 (60.4–104.7)	108.8 (82.1–124.5)	0.679

L, Leu; V, Val; X/V: Leu/Val and Val/Val.

SBP: systolic blood pressure, DBP: diastolic blood pressure; BMI: body mass index; WC: waist circumference; TG: triglycerides; TC: total cholesterol; cHDL: high density lipoprotein cholesterol; cLDL: low density lipoprotein cholesterol; FPG: fasting plasma glucose; FPI: fasting plasma insulin; HOMA-*β*%: %  *β*-cell function; HOMA-S%: % cell insulin sensitivity; HOMA-IR: insulin resistance; Cr: creatinine; UA: uric acid; GFR: glomerular filtration rate.

**Table 6:** Comparison of anthropometric, HOMA index, and biochemical characteristics of subjects between different groups of Pro12Ala PPAR*γ*2 genotypes in two subdivided groups according to age.

Parameters	Age < 60 years			Age ≥ 60 years		
	Pro12Ala PPAR*γ*2			Pro12Ala PPAR*γ*2		
	P/P (*n* = 289)	X/A (*n* = 125)	*P *	P/P (*n* = 80)	X/A (*n* = 28)	*P *
Age (years)	36 (29–46)	35 (28–41)	0.094	62 (59–66)	64 (59–69)	0.287
SBP (mmHg)	125 (120–140)	125 (120–130)	0.110	130 (120–140)	130 (120–140)	0.845
DBP (mmHg)	80 (80–85)	80 (80–80)	0.065	80 (80–85)	80 (75–90)	0.709
BMI (kg/m^2^)	26.0 (24.3–28.3)	25.4 (24.2–27.9)	0.276	26.9 (24.7–27.8)	25.9 (23.7–28.1)	0.237
WC (cm)	93.0 (84.0–98.0)	93.0 (82.5–97)	0.568	93.0 (86.0–98.0)	91.0 (79.0–97.5)	0.448
TG (mmol/L)	1.48 ± 0.71	1.44 ± 0.78	0.577	1.63 ± 0.70	1.34 ± 0.67	0.058
TC (mmol/L)	4.68 (4.11–5.45)	4.68 (4.10–5.25)	0.665	5.03 (4.46–5.68)	4.74 (4.10–5.57)	0.185
cLDL (mmol/L)	3.09 (2.61–3.71)	3.00 (2.54–3.66)	0.918	3.31 (2.92–3.84)	3.09 (2.82–3.84)	0.308
cHDL (mmol/L)	1.13 (0.88–1.28)	1.13 (0.91–1.34)	0.425	1.03 (0.88–1.24)	1.05 (0.87–1.41)	0.596
FPG (mmol/L)	6.08 ± 1.75	5.42 ± 1.49	<0.001	6.36 ± 1.84	5.58 ± 1.91	0.057
FPI (mIU/L)	9.74 (7.12–12.88)	7.92 (6.18–11.09)	<0.001	10.47 (7.30–12.89)	7.52 (6.01–11.49)	0.049
HOMA-*β*%	88 (66–101)	96 (82–106)	0.002	81 (55–99)	91 (82–103)	0.056
HOMA-S%	79 (55–110)	102 (68–126)	<0.001	73 (53–104)	103 (67–131)	0.030
HOMA-IR	1.3 (0.9–1.8)	1.0 (0.8–1.5)	<0.001	1.4 (1.0–1.9)	1.0 (0.8–1.5)	0.038
Cr (*μ*mol/L)	100.0 ± 45.4	89.3 ± 19.7	0.012	109.6± 47.1	107.0 ± 39.0	0.377
Urea (mmol/L)	6.15 ± 4.05	5.05 ± 2.19	0.005	7.54 ± 5.08	5.70 ± 2.90	0.073
UA (*μ*mol/L)	302 (247–387)	294 (241–353)	0.143	319 (248–383)	279 (211–347)	0.083
GFR (mL/min)	93.5 (83.3–109.4)	101.9 (85.2–112.9)	0.054	66.4 (48.9–77.6)	60.2 (52.7–71.6)	0.002

P: Pro; A: Ala; L: Leu; X/A: Pro/Ala and Ala/Ala.

SBP: systolic blood pressure, DBP: diastolic blood pressure; BMI: body mass index; WC: waist circumference; TG: triglycerides; TC: total cholesterol; cHDL: high density lipoprotein cholesterol; cLDL: low density lipoprotein cholesterol; FPG: fasting plasma glucose; FPI: fasting plasma insulin; HOMA-*β*%: %  *β*-cell function; HOMA-S%: % cell insulin sensitivity; HOMA-IR: insulin resistance; Cr: creatinine; UA: uric acid; GFR: glomerular filtration rate.

**Table 7:** Comparison of anthropometric, HOMA index, and biochemical characteristics of subjects between different groups of Leu162Val PPAR*α* genotypes in two subdivided groups according to age.

Parameters	Age < 60 years			Age ≥ 60 years		
	Leu162Val PPAR*α*			Leu162Val PPAR*α*		
	L/L (*n* = 345)	X/V (*n* = 69)	*P *	L/L (*n* = 96)	X/V (*n* = 12)	*P *
Age (years)	36 (28–45)	36 (28–43)	0.622	62 (60–67)	62 (60–69)	0.957
SBP (mmHg)	125 (120–140)	130 (120–135)	0.389	130 (120–140)	125 (120–135)	0.178
DBP (mmHg)	80 (80–85)	80 (80–85)	0.859	80 (80–85)	80 (75–80)	0.159
BMI (kg/m^2^)	25.7 (24.3–28.3)	26.6 (24.3–29.4)	0.147	26.9 (24.5–27.7)	26.8 (24.5–28.6)	0.965
WC (cm)	93.0 (82.0–97.0)	94.0 (87.5–98.5)	0.060	93.0 (85.5–97.5)	92.0 (81.5–98.5)	0.934
TG (mmol/L)	1.47 ± 0.73	1.47 ± 0.73	0.985	1.59 ± 0.69	1.24 ± 0.72	0.095
TC (mmol/L)	4.73 (4.19–5.49)	4.72 (4.14–5.42)	0.870	4.89 (4.48–5.68)	4.63 (3.95–5.27)	0.127
cLDL (mmol/L)	3.09 (2.61–3.71)	3.17 (2.57–3.64)	0.847	3.31 (2.91–3.84)	3.06 (2.39–3.79)	0.261
cHDL (mmol/L)	1.13 (0.88–1.29)	1.14 (0.92–1.34)	0.305	1.03 (0.88–1.27)	1.07 (0.76–1.33)	0.663
FPG (mmol/L)	5.96 ± 1.75	5.46 ± 1.33	0.026	6.34 ± 1.90	4.73 ± 0.84	0.005
FPI (mIU/L)	9.83 (7.12–12.88)	9.05 (6.78–12.28)	0.510	9.68 (7.12–12.89)	7.38 (5.54–11.04)	0.039
HOMA-*β*%	86 (64–100)	94 (77–111)	0.017	83 (56–99)	110 (84–131)	0.003
HOMA-S%	77 (54–109)	84 (61–118)	0.262	79 (53–111)	106 (72–142)	0.025
HOMA-IR	1.2 (0.9–1.8)	1.2 (0.9–1.7)	0.322	1.3 (0.9–1.9)	1.0 (0.8–1.4)	0.037
Cr (*μ*mol/L)	97.1 ± 40.7	95.3 ± 34.5	0.731	108.4 ± 44.0	113.3 ± 54.1	0.722
Urea (mmol/L)	5.99 ± 3.87	4.94 ± 1.82	0.028	7.36 ± 4.83	4.65 ± 1.94	0.058
UA (*μ*mol/L)	307 (248–385)	292 (233–341)	0.090	319 (237–377)	265 (217–352)	0.247
GFR (mL/min)	89.5 (72.7–105.1)	105.5 (91.8–117.2)	0.004	64.2 (47.1–75.1)	68.2 (56.9–82.1)	0.475

L, Leu; V, Val; X/V: Leu/Val and Val/Val.

SBP: systolic blood pressure, DBP: diastolic blood pressure; BMI: body mass index; WC: waist circumference; TG: triglycerides; TC: total cholesterol; cHDL: high density lipoprotein cholesterol; cLDL: low density lipoprotein cholesterol; FPG: fasting plasma glucose; FPI: fasting plasma insulin; HOMA-*β*%: %  *β*-cell function; HOMA-S%: % cell insulin sensitivity; HOMA-IR: insulin resistance; Cr: creatinine; UA: uric acid; GFR: glomerular filtration rate.

**Table 8:** Anthropometric and biochemical characteristics of subjects within different groups according to the simultaneous existence of different genotypes of the Leu162Val PPAR*α* and Pro12Ala PPAR*γ*2 polymorphisms.

	L/L P/P (*n* = 329)	L/L X/A (*n* = 112)	P/P X/V (*n* = 40)	X/A X/V (*n* = 41)	*P *
Age (years)	41 (29–54)	38 (28–49)	38 (29–47)	37 (28–49)	0.298
SBP (mmHg)	125 (120–140)	125 (120–140)	125 (120–140)	130 (120–135)	0.284
DBP (mmHg)	80 (80–85)	80 (80–85)	80 (80–90)	80 (75–80)	0.216
BMI (kg/m^2^)	26.4 (24.4–28.2)	24.9 (23.9–27.3)	26.0 (24.5–29.1	27.1 (24.2–29.1)	0.053
WC (cm)	93 (85–98)	93 (79–96)	93 (85–98)	95 (88–98)	0.052
TG (mmol/L)	1.53 ± 0.71	1.40 ± 0.75	1.38 ± 0.66	1.49 ± 0.79	0.289
TC (mmol/L)	4.73 (4.19–5.49)	4.71 (4.12–5.25)	4.73 (4.13–5.43)	4.71 (4.03–5.35)	0.645
cLDL (mmol/L)	3.13 (2.61–3.74)	3.00 (2.62–3.65)	3.06 (2.53–3.43)	3.20 (2.53–3.72)	0.578
cHDL (mmol/L)	1.09 (0.88–1.26)	1.13 (0.92–1.39)	1.17 (1.03–1.38)	1.05 (0.84–1.32)	0.028
FPG (mmol/L)	6.22 ± 1.80	5.55 ± 1.67	5.54 ± 1.32	5.18 ± 1.27	<0.001
FPI (mIU/L)	10.36 (7.12–12.89)	7.49 (6.02–10.93)	8.92 (6.95–12.44)	8.91 (6.16–11.26)	<0.001
HOMA-*β*%	84 (59–99)	94 (80–101)	89 (74–115)	99 (88–120)	<0.001
HOMA-S%	73 (54–108)	82 (58–117)	85 (59–115)	87 (67–128)	<0.001
HOMA-IR	1.4 (0.9–1.8)	1.2 (0.8–1.7)	1.2 (0.9–1.7)	1.1 (0.8–1.5)	<0.001
Cr (*μ*mol/L)	102.7 ± 46.2	90.4 ± 21.2	97.5 ± 42.9	98.4 ± 33.5	0.056
Urea (mmol/L)	6.61 ± 4.51	5.36 ± 4.54	5.15 ± 2.04	4.65 ± 1.59	<0.001
UA (*μ*mol/L)	313.0 (248.0–399.0)	301.5 (237.5–360.2)	304.0 (236.5–346.0)	266.0 (225.0–331.0)	0.017
GFR (mL/min)	87.9 (71.7–104.5)	91.5 (71.5–109.4)	101.5 (84.0–112.8)	102.6 (72.6–118.2)	0.011

P: Pro; A: Ala; L: Leu; V: Val; X/A: Pro/Ala and Ala/Ala; X/V: Leu/Val and Val/Val.

SBP: systolic blood pressure, DBP: diastolic blood pressure; BMI: body mass index; WC: waist circumference; TG: triglycerides; TC: total cholesterol; cHDL: high density lipoprotein cholesterol; cLDL: low density lipoprotein cholesterol; FPG: fasting plasma glucose; FPI: fasting plasma insulin; HOMA-*β*%: %  *β*-cell function; HOMA-S%: % cell insulin sensitivity; HOMA-IR: insulin resistance; Cr: creatinine; UA: uric acid; GFR: glomerular filtration rate.

## References

[1] Timar O, Sestier F, Levy E (2000). Metabolic syndrome X: a review. *Canadian Journal of Cardiology*.

[2] Liese AD, Mayer-Davis EJ, Tyroler HA (1997). Familial components of the multiple metabolic syndrome: the ARIC study. *Diabetologia*.

[3] Zatz R, Dunn BR, Meyer TW, Anderson S, Rennke HG, Brenner BM (1986). Prevention of diabetic glomerulopathy by pharmacological amelioration of glomerular capillary hypertension. *Journal of Clinical Investigation*.

[4] Cooper ME (1998). Pathogenesis, prevention, and treatment of diabetic nephropathy. *The Lancet*.

[5] Whelton PK, Perneger TV, He J, Klag MJ (1996). The role of blood pressure as a risk factor for renal disease: a review of the epidemiologic evidence. *Journal of Human Hypertension*.

[6] Chen J, Muntner P, Hamm LL (2004). The metabolic syndrome and chronic kidney disease in U.S. adults. *Annals of Internal Medicine*.

[7] Palaniappan L, Carnethon M, Fortmann SP (2003). Association between microalbuminuria and the metabolic syndrome: NHANES III. *American Journal of Hypertension*.

[8] Zhang L, Zuo L, Wang F (2007). Metabolic syndrome and chronic kidney disease in a Chinese population aged 40 years and older. *Mayo Clinic Proceedings*.

[9] Berger J, Moller DE (2002). The mechanisms of action of PPARs. *Annual Review of Medicine*.

[10] Berger JP, Akiyama TE, Meinke PT (2005). PPARs: therapeutic targets for metabolic disease. *Trends in Pharmacological Sciences*.

[11] Fajas L, Debril MB, Auwerx J (2001). Peroxisome proliferator-activated receptor-*γ*: from adipogenesis to carcinogenesis. *Journal of Molecular Endocrinology*.

[12] Mukherjee R, Jow L, Noonan D, McDonnell DP (1994). Human and rat peroxisome proliferator activated receptors (PPARs) demonstrate similar tissue distribution but different responsiveness to PPAR activators. *Journal of Steroid Biochemistry and Molecular Biology*.

[13] Guan Y, Zhang Y, Davis L, Breyer MD (1997). Expression of peroxisome proliferator-activated receptors in urinary tract of rabbits and humans. *American Journal of Physiology—Renal Physiology*.

[14] Ruan XZ, Moorhead JF, Fernando R, Wheeler DC, Powis SH, Varghese Z (2003). PPAR agonists protect mesangial cells from interleukin 1*β*-induced intracellular lipid accumulation by activating the ABCA1 cholesterol efflux pathway. *Journal of the American Society of Nephrology*.

[15] Guan Y, Zhang Y, Schneider A, Davis L, Breyer RM, Breyer MD (2001). Peroxisome proliferator-activated receptor-*γ* activity is associated with renal microvasculature. *American Journal of Physiology—Renal Physiology*.

[16] Alberti KGMM, Eckel RH, Grundy SM (2009). Harmonizing the metabolic syndrome: a joint interim statement of the International Diabetes Federation Task Force on Epidemiology and Prevention; National Heart, Lung, and Blood Institute; American Heart Association; World Heart Federation; International Atherosclerosis Society; and International Association for the Study of Obesity. *Circulation*.

[17] Wallace TM, Levy JC, Matthews DR (2004). Use and abuse of HOMA modeling. *Diabetes Care*.

[18] Cockcroft DW, Gault MH (1976). Prediction of creatinine clearance from serum creatinine. *Nephron*.

[19] Chung BH, Lim SW, Ahn KO (2005). Protective effect of peroxisome proliferator activated receptor gamma agonists on diabetic and non-diabetic renal diseases. *Nephrology*.

[20] Cuzzocrea S (2004). Peroxisome proliferator-activated receptors gamma ligands and ischemia and reperfusion injury. *Vascular Pharmacology*.

[21] Portilla D, Dai G, Peters JM, Gonzalez FJ, Crew MD, Proia AD (2000). Etomoxir-induced PPAR*α*-modulated enzymes protect during acute renal failure. *American Journal of Physiology—Renal Physiology*.

[22] Li S, Bhatt R, Megyesi J, Gokden N, Shah SV, Portilla D (2004). PPAR-*α* ligand ameliorates acute renal failure by reducing cisplatin-induced increased expression of renal endonuclease G. *American Journal of Physiology—Renal Physiology*.

[23] Deeb SS, Fajas L, Nemoto M (1998). A Pro^12^Ala substitution in PPAR*γ*
_2_ associated with decreased receptor activity, lower body mass index and improved insulin sensitivity. *Nature Genetics*.

[24] Sapone A, Peters JM, Sakai S (2000). The human peroxisome proliferator-activated receptor *α* gene: identification and functional characterization of two natural allelic variants. *Pharmacogenetics*.

[25] Stumvoll M, Stefan N, Fritsche A (2002). Interaction effect between common polymorphisms in PPAR*γ*
_2_ (Pro12Ala) and insulin receptor substrate 1 (Gly972Arg) on insulin sensitivity. *Journal of Molecular Medicine*.

[26] Koch M, Rett K, Maerker E (1999). The PPAR*γ*
_2_ amino acid polymorphism Pro 12 Ala is prevalent in offspring of Type II diabetic patients and is associated to increased insulin sensitivity in a subgroup of obese subjects. *Diabetologia*.

[27] Stefan N, Fritsche A, Häring H, Stumvoll M (2001). Effect of experimental elevation of free fatty acids on insulin secretion and insulin sensitivity in healthy carriers of the Pro12Ala polymorphism of the peroxisome proliferator-activated receptor-*γ*2 gene. *Diabetes*.

[28] Kurella M, Lo JC, Chertow GM (2005). Metabolic syndrome and the risk for chronic kidney disease among nondiabetic adults. *Journal of the American Society of Nephrology*.

[29] Chen J, Gu D, Chen CS (2007). Association between the metabolic syndrome and chronic kidney disease in Chinese adults. *Nephrology Dialysis Transplantation*.

[30] Raimundo M, Lopes JA (2011). Metabolic syndrome, chronic kidney disease, and cardiovascular disease: a dynamic and life-threatening triad. *Cardiology Research and Practice*.

[31] Perlstein TS, Gerhard-Herman M, Hollenberg NK, Williams GH, Thomas A (2007). Insulin induces renal vasodilation, increases plasma renin activity, and sensitizes the renal vasculature to angiotensin receptor blockade in healthy subjects. *Journal of the American Society of Nephrology*.

[32] Balkau B, Charles MA (1999). Comment on the provisional report from the WHO consultation. *Diabetic Medicine*.

[33] Khamaisi M, Flyvbjerg A, Haramati Z, Raz G, Wexler ID, Raz I (2002). Effect of mild hypoinsulinemia on renal hypertrophy: growth hormone/insulin-like growth factor 1 system in mild streptozotocin diabetes. *International Journal of Experimental Diabetes Research*.

[34] Wang S, DeNichilo M, Brubaker C, Hirschberg R (2001). Connective tissue growth factor in tubulointerstitial injury of diabetic nephropathy. *Kidney International*.

[35] Lupia E, Elliot SJ, Lenz O (1999). IGF-1 decreases collagen degradation in diabetic NOD mesangial cells: implications for diabetic nephropathy. *Diabetes*.

[36] Sjöholm A, Nyström T (2005). Endothelial inflammation in insulin resistance. *The Lancet*.

[37] Sowers JR (2007). Metabolic risk factors and renal disease. *Kidney International*.

[38] Kambham N, Markowitz GS, Valeri AM, Lin J, D’Agati VD (2001). Obesity-related glomerulopathy: an emerging epidemic. *Kidney International*.

[39] Bhowmik D, Tiwari SC (2008). Metabolic syndrome and chronic kidney disease.. *Indian Journal of Nephrology*.

[40] Hou X, Shen YH, Li C (2010). PPAR*α* agonist fenofibrate protects the kidney from hypertensive injury in spontaneously hypertensive rats via inhibition of oxidative stress and MAPK activity. *Biochemical and Biophysical Research Communications*.

[41] Calkin AC, Giunti S, Jandeleit-Dahm KA, Allen TJ, Cooper ME, Thomas MC (2006). PPAR-*α* and -*γ* agonists attenuate diabetic kidney disease in the apolipoprotein E knockout mouse. *Nephrology Dialysis Transplantation*.

[42] Bossé Y, Weisnage SJ, Bouchard C, Després JP, Louis Pérusse L, Vohl MC (2003). Combined effects of PPAR*γ*
_2_ P12A and PPAR*α*L162V polymorphisms on glucose and insulin homeostasis: the québec family study. *Journal of Human Genetics*.

[43] Jorsal A, Tarnow L, Lajer M (2008). The PPAR*γ*
_2_ Pro12Ala variant predicts ESRD and mortality in patients with type 1 diabetes and diabetic nephropathy. *Molecular Genetics and Metabolism*.

